# Effects of Low-Energy Diets Supplemented with *Lactobacillus reuteri* Postbiotic on Growth Performance and Intestinal Health of Broiler Chickens

**DOI:** 10.3390/ani16071011

**Published:** 2026-03-25

**Authors:** Meng Peng, Huiqin Sun, Wenhui Shi, Miaomiao Liu, Shuangshuang Guo, Dan Yi, Binying Ding, Mengjun Wu, Xiudong Liao, Giuseppe Maiorano, Peng Li

**Affiliations:** 1 Hubei Key Laboratory of Animal Nutrition and Feed Science, Wuhan Polytechnic University, Wuhan 430023, China; pengmeng33@gmail.com (M.P.); 13545801224@163.com (H.S.); 13507669796@139.com (M.L.); guo1shuangshuang@163.com (S.G.); yidan810204@163.com (D.Y.); dbying7471@126.com (B.D.); wumengjun93@163.com (M.W.); 2 Engineering Research Center of Feed Protein Resources of Agricultural By-Products, Ministry of Education, Wuhan Polytechnic University, Wuhan 430023, China; 3 Yichang Key Laboratory of Bio-Fermentation Engineering, Hubei Lan Good Microbial Technology Co., Ltd., Yichang 443100, China; h13298188579@163.com; 4 Mineral Nutrition Research Division, Institute of Animal Science, Chinese Academy of Agricultural Sciences, Beijing 100193, China; liaoxd56@163.com; 5 Department of Agricultural, Environmental and Food Sciences, University of Molise, 86100 Campobasso, Italy; maior@unimol.it

**Keywords:** low-energy diets, broilers, *Lactobacillus reuteri* postbiotic, growth performance, intestinal health

## Abstract

This study aims to investigate the effects of low-energy diets (LE) supplemented with *Lactobacillus reuteri* postbiotics (HSY) on growth performance and intestinal health of broiler chickens. A total of 2400 one-day-old Ross 308 broiler chicks with an average initial body weight of 46.10 ± 0.04 g were randomly assigned to a 2 × 2 factorial arrangement of treatments with 12 pens and 50 broiler chickens/pen for 39 days. Treatments were (1) CTR (basal diet), (2) LE (CTR-70 kcal ME/kg), (3) HSY (CTR + 0.5 kg/t HSY), and (4) LEHSY (LE + 0.5 kg/t HSY). LE increased the feed conversion ratio (FCR) of broilers (*p* = 0.03) without altering ADG, ADFI, and final BW. Supplementation with HSY significantly reduced the FCR of broilers (*p* = 0.001). HSY upregulated the activities of amylase and trypsin in jejunal digesta (*p* < 0.01). Furthermore, LE upregulated the expression of intestinal barrier-related genes such as *Mucin-2*, *Claudin-1* and *Occludin*, and HSY upregulated the expression of *Claudin-1* (*p* < 0.05). LE upregulated the expression of nutrient transport carriers such as *SGLT1* and *TRPV6* (*p* < 0.01), and HSY upregulated the expression of *TRPV6* (*p* < 0.01). LE upregulated the expression of immune-related genes such as *MHC-II* (*p* = 0.002), and HSY upregulated the expression of *IFN-γ, IL-10,* and *TGF-β* (*p* < 0.05). LE and HSY both downregulated the expression of intestinal lipid metabolism-related genes like *ACC*, while upregulating the expression of *FABP4* (*p* < 0.05). 16S rRNA sequencing showed that the HSY increased the Chao1 index of the jejunal microbiota and enriched beneficial bacteria such as *Lactobacillus salivarius* and *Lactobacillus avium*. LE and HSY both increased the concentrations of propionic and butyrate (*p* < 0.05). In summary, HSY can improve gut health and mitigate the negative impact of low-energy treatment on broiler growth performance by increasing the content of endogenous enzymes in the jejunum, improving gut microbiota structure, and increasing the content of short-chain fatty acids in the jejunum.

## Simple Summary

Lowering dietary energy reduces feed costs but may impair intestinal function and growth performance. In the context of antibiotic-free production systems, the development of effective functional feed additives that can sustain intestinal homeostasis under nutritional stress is of considerable importance. In this study, we investigated whether supplementation with a *Lactobacillus reuteri*-derived postbiotic could mitigate the negative impacts of low-energy diets on broiler chickens. Our findings suggest that *Lactobacillus reuteri*-derived postbiotic alleviates the adverse effects of energy restriction by enhancing endogenous digestive enzyme activity in the jejunum, reshaping the intestinal microbial community and promoting the production of short-chain fatty acids, thereby improving gut health and supporting growth performance.

## 1. Introduction

Optimizing broiler gut health through green and safe nutritional strategies, thereby comprehensively improving production efficiency, remains a pivotal challenge facing the modern poultry industry. Probiotics play a crucial regulatory role in the intestinal microbial ecosystem of poultry, contributing to maintaining gut environment stability and resilience [1]. *Lactobacillus reuteri*, a naturally occurring heterofermentative lactic acid bacterium in the intestines, secretes multiple bioactive metabolites, including reuterin, extracellular polysaccharides, and organic acids to improve gut health [2]. Extensive studies have confirmed that *Lactobacillus reuteri* effectively modulates the balance of the gut microbiota, thereby exerting multiple physiological functions, including eliminating specific intestinal infections and alleviating colonic inflammation [3]. However, probiotics, being live microorganisms, face inherent limitations in application. On one hand, they are prone to inactivation due to environmental factors during production, storage, and transportation, leading to reduced efficacy [4]. On the other hand, they may harbor resistance genes or virulence factors, posing potential safety risks [5].

Postbiotics are defined as mixtures of inactivated probiotics and their metabolic products [6]. By eliminating live bacteria, postbiotic products fundamentally avoid risks associated with live cultures, such as bacteremia and fungemia [7]. Additionally, postbiotics possess excellent tolerance to temperature, pH, and various processing techniques [8]. Thus, postbiotics deliver some probiotic benefits while avoiding their drawbacks. Previous studies revealed that *Lactobacillus reuteri* postbiotics (HSY) exhibit significant antibacterial and antioxidant properties, suppressing the proliferation of common foodborne pathogens such as *Escherichia coli* and *Staphylococcus aureus* [9]. This action plays a crucial role in maintaining the stability of the structure and composition of the gut microbiota [10]. Our recent findings indicate that HSY supplementation in the diet enhances the growth performance and intestinal health of broilers under necrotic enteritis challenge, partly through improvements in intestinal morphology [11]. However, this study has some limitations, such as the small number of animal replicates (180 animals) and the lack of research on the effects of HSY on the entire life cycle of broilers.

The scaling and intensification of broiler farming have led to feed resource shortages and insufficient feed grain supply, posing significant challenges to the broiler industry [12]. Low-energy diet technology, as an innovative feeding strategy, is highly regarded for its combined economic and environmental benefits [13]. The core of this technology lies in implementing precision nutrition programs: by scientifically adjusting the energy density and nutritional structure of diets, it significantly reduces reliance on high-energy ingredients like corn and oils while ensuring basic growth requirements are met [14]. A previous study found that low-metabolizable-energy diets have no effect on the growth performance or carcass yield of broilers; however, feeding low-metabolizable-energy diets (3080 kcal/kg) can affect the weight of broiler organs and small intestines, as well as meat quality characteristics [15]. Research indicates that low-energy diets (3124 kcal/kg, metabolizable energy) activate the AMPK signaling pathway within the body, enhancing energy utilization efficiency and suppressing muscle catabolism [16]. Furthermore, by reducing the use of traditional staple ingredients like corn and soybean meal, low-energy diets (3824 kcal/kg, gross energy) help minimize the excretion of nutrients such as nitrogen and phosphorus at the source, effectively alleviating the eutrophication pressure on aquatic environments caused by the livestock industry [17]. Conversely, one study reported that reduced dietary energy levels (2950 kcal/kg, metabolizable energy) decrease the abundance and diversity of microbial species in the cecum of broilers, impairing their immune function [18]. The reasons for these differences may be numerous, such as genotype, feed formulation/processing methods, and husbandry practices. Therefore, further research is needed on the effects of low-energy diets on broiler growth performance and intestinal function.

Low-energy diets offer positive effects in improving broiler production levels and farming profitability, alleviating feed resource shortages, and reducing environmental emissions. HSY also shows promising potential in broiler farming. However, whether combining these approaches synergistically promotes healthy and efficient broiler farming remains unknown. The objective of the present study was to evaluate the effects of HSY supplementation in low-energy diets on growth performance and intestinal health in broiler chickens.

## 2. Materials and Methods

### 2.1. Preparation of Lactobacillus reuteri Postbiotics

The preparation of *Lactobacillus reuteri* postbiotics was employed according to our previous method [11]. In short, the *L. reuteri* (RZ-D15L14) was isolated from the intestinal mucosa of poultry for culture, amplification, and identification. The above isolated *L. reuteri* was inoculated into MRS medium under anaerobic fermentation conditions. Then, 10% maltodextrin was supplemented to the fermentation broth for adsorption, and finally, the *L. reuteri* postbiotics (HSY) were produced through spray-drying.

### 2.2. Animal Experiment

The animal experiment was carried out at the broiler research facility of Wuhan Polytechnic University (Wuhan, Hubei Province, China). A total of 2400 one-day-old Ross 308 broiler chicks (equal numbers of males and females) with an average initial body weight of 46.10 ± 0.04 g were used in this study. Birds were randomly distributed into a 2 × 2 factorial experimental design with 12 replicate pens per treatment and 50 birds per pen. The chicks were assigned to the following dietary treatments: (i) negative control group (CTR group, basal diet); (ii) low-energy-diet group (LE, CTR-70 kcal ME/kg); (iii) *Lactobacillus reuteri* postbiotics group (HSY, basal diet plus 0.5 kg/t HSY); and (iv) low-energy diet supplemented with *Lactobacillus reuteri* postbiotics group (LEHSY, LE plus 0.5 kg/t HSY). All chickens were raised in wire cages with a size of 2 m × 4 m (6.25 chicks/m^2^), and a 24 h light regime was implemented, according to the routine management programs of Ross 308 broilers. Feed and water were provided ad libitum using plastic feeders and plastic water troughs, which were manually refilled to ensure continuous availability throughout the experiment. For the first 3 days of the experiment, the room temperature was controlled at 33 °C, and then decreased by 2 °C each week until it was maintained at 24 °C. The basal diets were formulated according to the recommendation of the Chinese chicken feeding standard (NY/T 33-2004) [19], as shown in Table 1. The trial lasted 39 days, with two phases of feeding: from day 1 to day 14, a basal diet was given to all groups for adaptation, while from day 15 to day 39, a customized diet was supplied for each group. All broilers were weighed at day 1, day 14, and day 39 of age after a 12 h fasting. Average daily gain (ADG), average daily feed intake (ADFI), feed conversion ratio (FCR), and European performance efficiency factor (EPEF) were calculated [20]. All animal procedures in this study were approved by the Institutional Animal Care and Use Committee of Wuhan Polytechnic University (Approval No.: WPU202310006). The experiments were performed in accordance with the Animal Research: Reporting of In Vivo Experiments (ARRIVE) guidelines.

### 2.3. Sample Collection

On day 39, twelve chickens per group were randomly selected, and blood was collected from the wing vein, followed by euthanasia by cervical dislocation [21]. After centrifugation (4 °C, 3000× *g*, 15 min), the serum was collected for subsequent analysis. Anatomical measurements were performed in a BSL-2 biosafety laboratory by using an electronic digital caliper. The lengths of the duodenum (from the pyloric sphincter to the opening of the pancreatic duct), jejunum (from the pancreatic duct to the yolk sac diverticulum), ileum (from the yolk sac diverticulum to the ileocecal ligament), and cecum (from the ileocecal ligament to the cecal tip) were measured by gently flattening the tissue along the contralateral mesentery. Then, a 1 cm section of tissue was taken from the mid-jejunum and immediately fixed in 4% neutral buffered paraformaldehyde (pH 7.4, pre-cooled to 4 °C). Simultaneously, jejunal chyme was collected and stored at −80 °C for microbial composition analysis [22].

### 2.4. Plasma Biochemical Indices

Total protein (TP), albumin (ALB), glucose (GLU), uric acid (UA), triglycerides (TG), alanine aminotransferase (ALT), aspartate aminotransferase (AST), creatinine (CRE), creatine kinase (CK), alkaline phosphatase (ALP), lactate dehydrogenase (LDH), serum calcium (CA), and phosphorus (IP) were measured using a fully automated biochemical analyzer (HITEC 7100, Hitachi, Ltd., Tokyo, Japan). The kits were purchased from Shanghai Huake Biotechnology Co., Ltd. (Shanghai, China).

### 2.5. Observation of Intestinal Morphology

First, jejunal samples fixed with 4% paraformaldehyde were processed using a standardized procedure at Wuhan Boster Biological Engineering Co., Ltd. (Wuhan, China), including gradient ethanol dehydration, paraffin embedding (60 °C ± 2 °C), 4 μm serial section preparation, and HE double staining (hematoxylin staining for 5 min, eosin counterstaining for 2 min). Then, micromorphometric analysis was performed using an OLYMPUS BX-41TF microscopic imaging system (equipped with a DP27 digital camera) (Olympus, Tokyo, Japan), focusing on quantifying three key morphological parameters: vertical distance from the villus height to the base (VH), depth from the base of the crypt to the crypt opening (CD), and the ratio of the two (V/C value). Five complete villus–crypt units were selected randomly from each section for three repeated measurements. The final data were standardized using ImageJ 1.53t software (National Institutes of Health, Bethesda, MD, USA).

### 2.6. RNA Isolation and Quantitative Real-Time PCR

Total RNA was isolated from jejunal samples using RNAiso Plus reagent (Takara, Dalian, China). cDNA was synthesized using the PrimeScript reagent kit with gDNA Eraser (Takara, Dalian, China). Quantitative real-time PCR (qRT-PCR) analysis was conducted on an ABI 7500 Fast Real-Time PCR System (Applied Biosystems, Foster City, CA, USA) following the manufacturer’s protocol [11,23]. The relative expression levels of target genes were determined using the 2^−ΔΔCt^ method with β-actin serving as the reference gene. All primers were synthesized by Sangon Biotech Co., Ltd. (Shanghai, China), and their sequences are presented in Table 2.

### 2.7. Determination of Endogenous Enzyme Content in Jejunal Contents

The activities of endogenous digestive enzymes in the jejunal contents were measured using commercial assay kits purchased from Nanjing Jiancheng Bioengineering Institute (Nanjing, China). In detail, lipase catalytic activity in jejunal contents was determined using a colorimetric method (catalog number A054-2-1), α-amylase hydrolysis efficiency was analyzed using the starch–iodine colorimetric technique (catalog number C054-2-1), and trypsin activity was assessed using the casein substrate method (catalog number A080-2). All assay procedures were strictly performed in accordance with the manufacturer’s instructions.

### 2.8. Short-Chain Fatty Acid Determination

Jejunal content samples (50 mg) were thawed at room temperature and mixed with 1.5 mL of sterile demineralized water, then homogenized twice for 120 s to ensure thorough mixing. After 30 min balance, the sample was centrifuged at 15,000 r/min for 20 min. Then, 1 mL of the supernatant was mixed with 0.2 mL of 25% (*m*/*v*) metaphosphoric acid solution (*v*:*v* = 5:1). The mixture was incubated at 4 °C for 30 min, then centrifuged at 15,000 r/min for 20 min, followed by filtering the supernatant through a 0.22 μm filter membrane. Finally, the content of acetic acid, propionic acid, and butyric acid were analyzed using an Agilent 7980B gas chromatograph (Agilent Technologies, Palo Alto, CA, USA) [24].

### 2.9. Jejunal Microbiota Analysis

The separation and purification of microbial DNA from the jejunum contents were conducted following the instructions of the QIAamp DNA Stool Mini Kit (Qiagen, Valencia, CA, USA). To accurately assess nucleic acid quality, the obtained DNA was determined using a NanoDrop^®^ND-1000A UV-VIS spectrophotometer (Thermo Scientific, Wilmington, DE, USA). Subsequently, the purity of the DNA samples was tested by 1% agarose gel electrophoresis. Qualified DNA samples were amplified targeting the V3–V4 region of the bacterial 16S rRNA gene using universal primers 338F (5′-ACTCCTACGGAGGCAGCA-3′) and 806R (5′-GGACTACHVGGGTWTCTAAT-3′). The resulting PCR amplicons were purified, quantified, and pooled to construct sequencing libraries using the TruSeq DNA PCR-Free Sample Preparation Kit (Illumina, San Diego, CA, USA). The libraries were subsequently sequenced on an Illumina HiSeq PE250 platform (Illumina, CA, USA). Species abundance tables at different taxonomic levels were generated using the Qiime2-2019.7 pipeline [25], and the α-diversity of samples was analyzed. LEfSe analysis was then performed to identify statistically significant biomarkers between groups based on LDA values. R software (Version 2.15.3) was used to generate data graphs and perform ANOSIM analysis. The raw sequencing data have been deposited in the NCBI Sequence Read Archive under accession number PRJNA1414861.

### 2.10. Statistical Analysis

All data were analyzed using SPSS 20.0 statistical software (SPSS Inc., Chicago, IL, USA). One-way ANOVA was used to analyze the growth performance of chicks aged 1–14 days. Other data were analyzed using a two-way ANOVA with diet energy level and postbiotic supplementation as fixed factors. Duncan’s multiple range test was applied when there was a significant interaction between groups. The experimental results are expressed as means ± SEM, and the significance of the main effect and interaction was also provided. *p* < 0.05 was considered statistically significant between groups, while *p* < 0.01 indicated highly significant differences.

## 3. Results

### 3.1. Effects of Low-Energy Diets and HSY on Broiler Growth Performance

There were no differences in BW at d 14, as well as in ADG, ADFI, and FCR among broilers from d 1 to d 14 (*p* > 0.05), as shown in Table 3. From d 15 to d 39, energy and HSY had an interactive effect on the FCR of broilers (*p* < 0.05). Compared with the control group, reducing energy significantly increased the FCR, while adding HSY to a low-energy diet significantly decreased the FCR (*p* < 0.05). Energy and HSY had no effect on ADFI or ADG, as shown in Table 4. From d 1 to d 39, compared with the control group, a low-energy diet significantly increased the FCR (*p* < 0.05), while HSY significantly decreased the FCR (*p* < 0.05). Energy and HSY had no difference in BW at d 39, ADFI from d 1 to d 39, and ADG, as shown in Table 5. In addition, Table 6 shows that LE significantly reduced the EPEF of broilers at 21, 28, and 39 days of age (*p* < 0.05) while HSY significantly reduced the EPEF at 21 days of age (*p* < 0.05).

### 3.2. Effects of Low-Energy Diets and HSY on Plasma Biochemical Parameters of Broilers

LE significantly increased serum AST and CK levels (*p* < 0.05), while HSY significantly decreased serum P, DB, and LDH levels (*p* < 0.05) and significantly increased serum GGT levels (*p* < 0.05), as shown in Table 7 (effects of HSY and LE on serum biochemical parameters of broilers). Furthermore, there was an interaction effect between LE and HSY on ALP levels (*p* < 0.05). Compared to the control group, LE did not affect serum ALP levels (*p* < 0.05), but HSY supplementation significantly reduced serum ALP levels under LE conditions (*p* < 0.05).

### 3.3. Effects of Low-Energy Diets and HSY on Intestinal Index and Intestinal Morphology of Broilers

**Figure 1:**
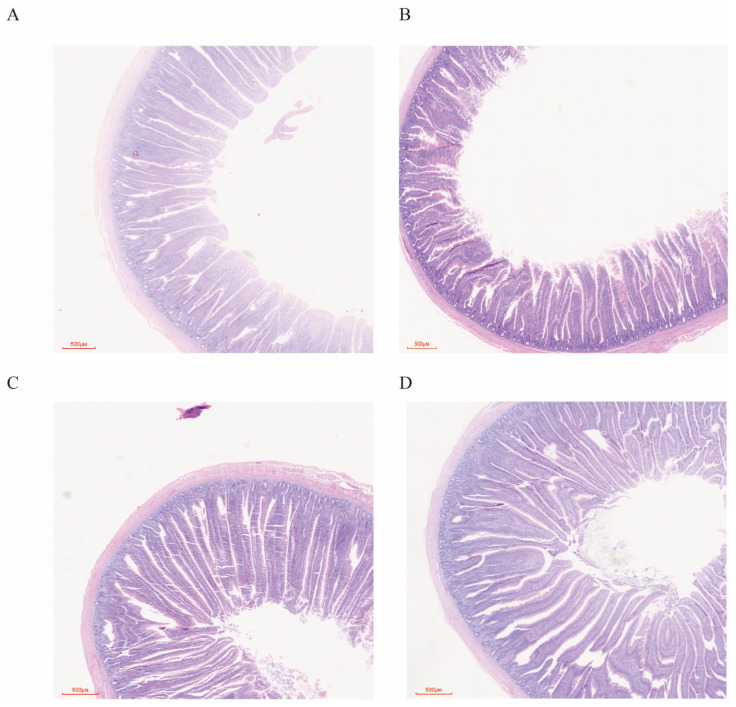
Effects of LE and HSY on intestinal morphology of broilers. (**A**): CTR (basal diet), (**B**): LE (CTR-70 kcal ME/kg), (**C**): HSY (CTR + 0.5 kg/t HSY), (**D**): LEHSY (LE + 0.5 kg/t HSY).

As shown in Table 8, LE and HSY had no difference in the intestinal length index of broilers (*p* > 0.05). LE significantly reduced the jejunal villus surface area of 39-day-old broilers (*p* < 0.05), while HSY significantly reduced the ratio of villus height to crypt depth (*p* < 0.05). However, there was no difference between LE and HSY on villus height and crypt depth in broilers (*p* > 0.05), as indicated by Table 9 and Figure 1.

### 3.4. Effects of HSY and LE on the mRNA Relative Expression of the Intestinal Mucosal Barrier, Nutrient Transport, Immunity, and Lipid Metabolism Genes of Broilers

LE upregulated *Mucin-2* expression, while HSY downregulated *Mucin-2* expression (*p* < 0.05). LE and HSY had interactions on *Claudin-1* and *Occludin* expression (*p* < 0.05). Compared with the control group, LE upregulated the expression levels of both *Claudin-1* and *Occludin* (*p* < 0.05); HSY addition upregulated the relative expression level of *Claudin-1* (*p* < 0.05) and downregulated the relative expression level of *Occludin* (*p* < 0.05) under LE conditions, as shown in Table 10.

There was an interaction between LE and HSY on the relative expression of *SGLT1, TRPV6, KCNJ13, SLC1A1*, and *pepT1* (*p* < 0.05). Compared with the control group, LE upregulated the expression of *SGLT1* and *SLC1A1* (*p* < 0.05) and downregulated the expression of *TRPV6* (*p* < 0.05). HSY addition upregulated the expression of *TRPV6* (*p* < 0.05) and downregulated the expression of *SGLT1* (*p* < 0.05), under LE conditions.

LE upregulated the relative expression of *MHC-II* in the jejunum of broilers (*p* < 0.05), while HSY upregulated the relative expression of *IFN-γ* and *TGF-β* (*p* < 0.05). LE and HSY interacted on the relative expression of the immune genes IL-10 and *IL-1β* in the jejunum of broilers (*p* < 0.05). Compared with the control group, LE downregulated the relative expression of *IL-10* and *IL-1β*, while HSY upregulated the relative expression of *IL-10* and *IL-1β* (*p* < 0.05) under LE conditions.

LE downregulated the relative expression of *ACC* and *SREBF1* in the broiler intestine (*p* < 0.05) and upregulated the relative expression of *FABP4* (*p* < 0.05), while HSY downregulated the expression of *LXRα* (*p* < 0.05) and upregulated the expression of *FABP4* (*p* < 0.05). LE and HSY had an interaction on the expression of *ACOT8* (*p* < 0.05). Compared with the control group, LE upregulated the expression of *ACOT8* (*p* < 0.05), and the addition of HSY under the condition of LE downregulated the expression of *ACOT8* (*p* < 0.05).

### 3.5. Effects of HSY and LE on the Jejunal Short-Chain Fatty Acids and Lysozyme of Broilers

Both LE and HSY increased the content of propionic acid and butyric acid in the jejunal contents of broilers (*p* < 0.05). LE and HSY had an interactive effect on the content of propionic acid in the jejunal contents (*p* < 0.05). Compared with the control group, LE increased the propionic acid content (*p* < 0.05), while the addition of HSY to LE decreased the propionic acid content (*p* < 0.05), as indicated by Table 11. In addition, HSY increased the content of jejunal α-amylase and trypsin of broilers (*p* < 0.05), while LE and HSY had no effect on the lipase content in broiler jejunal contents (Table 12).

### 3.6. Effects of HSY and LE on the Jejunal Microbiota of Broilers

As shown in Table 13, HSY increased the Chao1 index. LE and HSY had no effect on the Simpson and Shannon indices (*p* > 0.05).

**Figure 2:**
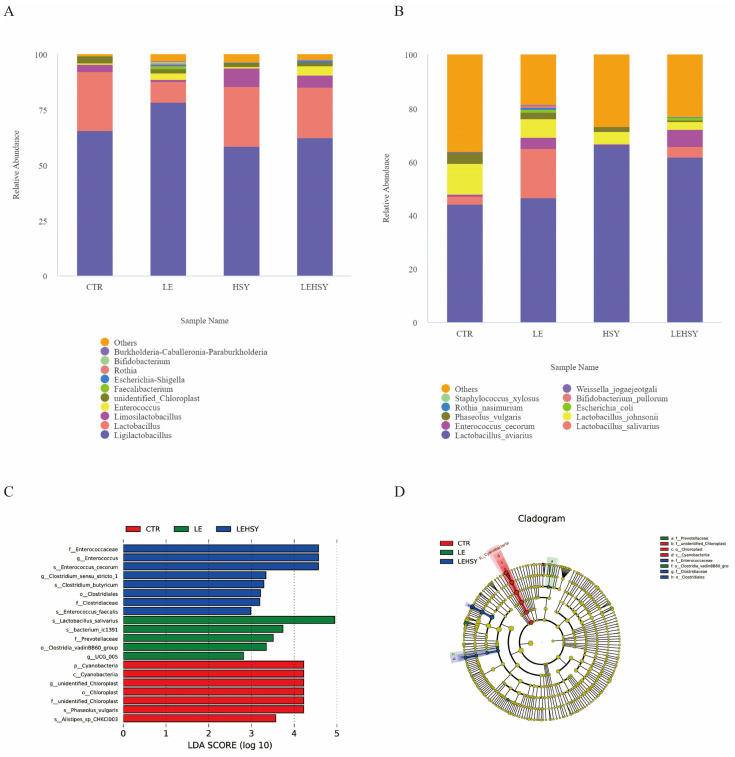
Effects of HSY and LE on the jejunal microbiota of broilers. (**A**): Differential species at the phylum level, (**B**): differential species at the genus level, (**C**): LDA analysis, (**D**): cladogram analysis.

At the genus level (Figure 2A), the dominant gut microbiota in the CTR group of broilers were *Lactobacillus* and *Ligilactobacillus*. Compared to the CTR group, LE increased the relative abundance of *Ligilactobacillus, Enterococcus, and Faeacalibacterium*, and decreased the relative abundance of *Lactobacillus* and *Unidentified-Chloroplast*. HSY decreased the relative abundance of *Limosilactobacillus* and *Unidentified-Chloroplast*, increased the relative abundance of *Enterococcus*. Compared to the LE group, the trend of decreasing *Lactobacillus* levels was improved in the LESHY group. At the species level (Figure 2B), compared to the CTR group, LE increased the abundance of *Lactobacillus salivarius* and decreased the relative abundance of *Lactobacillus johnsonii*. HSY increased the abundance of *Lactobacillus salivarius* and *Lactobacillus aviarius* within the *Lactobacillus* genus, while the LESHY group increased the abundance of *Enterococcus cecorum* and *Lactobacillus salivarius*.

Specifically, the LDA bar chart results showed that at the species level, the dominant bacterial group in the LE group was *Lactobacillus salivarius*, and at the family level it was *Prevostaceae* (Figure 2C). At the family and genus level, the dominant bacterial group in the LEHSY group was *Enterococci*, and at the species level, it was *Enterococcus cecorum* and *Clostridium butyricum*. The phylogenetic tree analysis showed that the species richness of the control group was higher than that of the LE group and the LEHSY group (Figure 2D).

## 4. Discussion

Low-energy diets offer positive effects in improving broiler production levels and farming profitability, alleviating feed resource shortages, and reducing environmental emissions. *Lactobacillus reuteri* postbiotics (HSY) exhibit significant antibacterial and antioxidant properties, showing promising potential in broiler farming. Therefore, this study investigated the effects of low-energy diets supplemented with HSY on the growth performance and intestinal health of broiler chickens. The results suggested that HSY can improve gut health and mitigate the negative impact of low-energy treatment on broiler growth performance by increasing the content of endogenous enzymes in the jejunum, improving gut microbiota structure, and increasing the content of short-chain fatty acids in the jejunum.

### 4.1. HSY Improves Feed Utilization in Broilers Fed with LE Diet

Broiler chickens grow rapidly and have active metabolisms, which require significant energy for tissue and muscle development. While energy is insufficient, the body preferentially breaks down fat for energy, resulting in a lower carcass fat percentage and reduced protein synthesis, which delays muscle development. Previous studies have shown that reducing the metabolizable energy of broiler diets from 3200 kcal/kg to 2800 kcal/kg can lead to a 10–15% decrease in body weight at 42 days of age [26]. Conversely, the present study shows that although reducing LE by only 70 kcal/kg increased the feed conversion ratio (FCR) of broilers, the diet did not affect final body weight (BW), average feed intake index (ADFI), and average feed growth rate (ADG). This indicates that slightly reducing the energy status of the diet does not affect broiler growth performance. Interestingly, adding HSY to LE diets significantly reduced the FCR in broilers aged 1–39 days and 15–39 days, suggesting it may mitigate the increase in feed intake caused by energy reduction in the diet, which may be the main reason for its improved feed conversion efficiency. Similarly, previous studies have found that *Lactobacillus reuteri* helps increase broiler weight and improve feed conversion ratio [27]. Another study found that supplementation with HSY markedly enhanced average daily gain (ADG) relative to the E. coli-challenged group during days 1–18, while average daily feed intake (ADFI), feed conversion ratio (FCR), and mortality were not significantly affected by the treatments [28]. The study has reported that postbiotic supplementation improves growth performance under pathogenic stress conditions, such as E. coli challenge. The present study demonstrates that similar beneficial effects also occur under nutritional stress induced by a low-energy diet, suggesting that postbiotics may exert positive roles across different stress conditions. We hypothesize that *Lactobacillus reuteri* postbiotics may improve feed utilization in broilers under low-energy models by enhancing gut health.

### 4.2. HSY Improves Plasma Biochemical Indices in Broilers Fed with LE Diet

Serum biochemical indicators of broiler chickens are an important basis for assessing their health status and feeding management effects. This study found that reducing energy significantly increased the levels of AST and CK in broiler serum. AST and CK are sensitive markers of cell damage. Elevated AST may be associated with liver damage [29], while increased CK usually reflects skeletal muscle or myocardial damage [30]. Insufficient energy intake may lead to increased catabolism, muscle protein degradation, or increased oxidative stress, thereby releasing more AST and CK [31]. In addition, decreased LDH levels may be associated with increased cell membrane stability [32]. This study also found that HSY significantly reduced the levels of P, DB, and LDH in serum. This result suggests that reduced energy may produce a stress response in liver and muscle tissue, and that HSY may have a mitigating effect to maintain body homeostasis. However, a previous study suggested dietary HSY has no significant impact on most plasma biochemical indices in coccidia- and Clostridium perfringens-challenged birds [11]. This suggests that the sensitivity of HSY may vary in different animal models.

### 4.3. Effects of LE and HSY on Intestinal Barrier Function and Nutrient Transport in Broilers

Intestinal villus height, crypt depth, and villus surface area are important indicators for evaluating intestinal barrier function. The results of the present study show that LE significantly reduced the villus surface area of broiler jejunum. One previous study has shown that insufficient energy supply reduced ATP production in intestinal mucosal cells, inhibited mTOR signaling pathway activity, and thus reduced the proliferation rate of villus epithelial cells [33]. Another research also found that low-energy diets lead to a reduction in the width of the jejunum villi in broiler jejunum [34], which is basically consistent with the results of the decrease in villus surface area in this study. In contrast, LE and HSY did not affect the villus height and crypt depth of broiler intestinal jejunum. Similarly, another study reported that HSY did not affect the histologic score of the jejunum. Although HSY supplementation significantly reduced the villus-height-to-crypt-depth ratio, the individual parameters (villus height, crypt depth, and villus area) were not significantly affected. Therefore, the biological significance of this change should be interpreted cautiously. In addition, HSY supplementation increased digestive enzyme activities and no obvious intestinal damage was observed in histological sections, suggesting that intestinal function was not impaired. Jejunal barrier-related genes directly affect the integrity of the mechanical barrier by regulating the connection structure between intestinal epithelial cells. The present study found that low energy upregulates the expression of *Claudin-1* and *Occludin*, indicating that energy restriction may upregulate the expression of tight junction proteins by enhancing barrier repair capacity [35]. Furthermore, previous studies have found that HSY may activate Claudin-1 transcription through SCFAs [36] and may also optimize barrier selective permeability by regulating the stability of the *ZO-1*/*Occludin* complex [37]. Similarly, the present study indicated that HSY increased the expression of *Claudin-1* in the jejunum of broilers. In addition, studies have reported that reduced energy intake may compensate for insufficient energy intake by enhancing the active transport of glucose (*SGLT1*) and glutamate (*SLC1A1*) through the *GLP-2* or *PKA* signaling pathways [38]. Similarly, this study also observed that low energy intake upregulated the expression of *SGLT1/SLC1A1*. Conversely, postbiotics may reduce glucose absorption by inhibiting *SGLT1* expression, thereby promoting the competitive utilization of carbohydrates by gut microbiota [39]. *TRPV6* is mainly involved in mediating calcium ion absorption. This study found that low-energy treatment downregulated the expression of *TRPV6* in the gut, but HSY could alleviate the downregulation of *TRPV6* expression caused by reduced energy intake. In conclusion, LE and HSY may enhance intestinal barrier function and nutrient transport in the jejunum of broilers.

### 4.4. Effects of LE and HSY on Immunity and Lipid Metabolism in Broilers

Low-energy diets inhibit the normal expression of intestinal immune function in broilers through multiple pathways, such as increased levels of pro-inflammatory cytokines. In contrast, bioactive components in postbiotics (including antimicrobial peptides, extracellular polysaccharides, and short-chain fatty acids) can exert immunomodulatory effects by regulating immune cell function. The results of this study showed that HSY supplementation significantly upregulated the gene expression levels of interferon-γ (IFN-γ) and transforming growth factor-β (TGF-β) in the jejunum. IFN-γ, as a key mediator of Th1 immune response, works synergistically with TGF-β, which has immunomodulatory functions, to maintain intestinal immune homeostasis [40]. At the same time, the combined intervention of LE and HSY led to the simultaneous downregulation of interleukin-10 (IL-10) and interleukin-1β (IL-1β) expression, indicating that the excessive inflammatory response was effectively controlled. Similarly, previous studies have found higher levels of IL-10 mRNA expression in the jejunal and ileal mucosa of HSY, and higher levels of TGF-β mRNA expression in the jejunal mucosa [28]. Furthermore, compared to the CTR group, the HSY group also showed increased mRNA levels of IFN-γ, IL-10, TGF-β, and Foxp3 genes in the jejunum of birds [11]. Notably, energy restriction significantly increased the expression levels of major histocompatibility complex class II (MHC-II) molecules, suggesting enhanced antigen-presenting capacity of the jejunal mucosa.

Postbiotics, as metabolites of probiotics, have been shown to indirectly regulate the expression of lipid metabolism-related genes. Fatty acid-binding protein 4 (FABP4) and acetyl-CoA carboxylase (ACC) are key regulators of lipid synthesis and transport. This study found that LE induced upregulation of *FABP4* and *ACC* expression, which may be an adaptive response of the body to enhance fatty acid uptake and synthesis to compensate for energy deficiency. HSY significantly reduced *FABP4* and *ACC* expression while upregulating sterol regulatory element-binding protein 1 (*SREBF-1*). This regulatory pattern suggests that metabiotic may reduce peripheral lipid deposition and inhibit ACC activity by inhibiting fatty acid transport (through downregulation of *FABP4*), thereby preventing excessive lipogenesis [41,42]. Furthermore, LE significantly upregulated *LXRα* expression, while HSY decreased both *LXRα* and *ACOT-8* expression. In summary, this study indicates that low-energy diets induce lipid metabolism stress, while metabiotic effectively maintain intestinal lipid metabolism homeostasis in broilers.

### 4.5. Effects of HSY and LE on the Jejunal Short-Chain Fatty Acids and Lysozyme of Broilers

Short-chain fatty acids (SCFAs), including acetic acid, propionic acid, and butyric acid, are important bioactive substances produced by gut microbiota metabolism and play a key role in maintaining gut homeostasis, immune regulation, and anti-inflammatory processes [43]. Acetic acid is mainly produced by Lactobacillus and can regulate gut pH and promote microbial growth, but excessive amounts can induce the expression of pro-inflammatory factors IL-1β and IL-6 [44]. Propionic acid can enhance intestinal barrier function by promoting the secretion of anti-inflammatory factors and upregulating the expression of ZO-1 [45]. Butyric acid, as the main energy source for intestinal epithelial cells, is crucial for maintaining barrier integrity [46]. In this study, low-energy diets significantly increased the content of propionic acid and butyric acid in jejunal contents. This change may be due to the increased accumulation of undigested carbohydrates (such as resistant starch and dietary fiber) in the hindgut of the LE-diet mice, providing sufficient substrate for microbial fermentation. At the same time, low-energy diets may promote butyric acid synthesis by increasing the Firmicutes/Bacteroidetes ratio [47] and prolonging the retention time of chyme in the intestine, thereby enhancing fermentation efficiency [48]. The addition of HSY further increased the levels of propionic and butyric acids, which may be related to the direct promotion of acid-producing bacteria proliferation by its inactivated bacterial components (such as peptidoglycan and extracellular polysaccharides). These findings are consistent with previous studies that probiotic supplementation can significantly increase the concentration of SCFAs in the gut [49].

The levels of intestinal digestive enzymes (such as proteases, amylases, and lipases) are key indicators for assessing the efficiency of nutrient digestion in livestock and poultry. This study found that the addition of HSY to low-energy diets significantly increased the activity of amylase and trypsin in jejunal content but had no significant effect on lipase levels. Previous studies have found that inactivated bacterial components (such as peptidoglycan and extracellular polysaccharides) in metabiotics may stimulate pancreatic exocrine function by activating Toll-like receptors (TLRs) in intestinal epithelial cells, thereby promoting the secretion of amylase and trypsin [50]. In addition, short-chain fatty acids (SCFAs) produced by postbiotics can enhance pancreatic secretion by activating G protein-coupled receptors (GPCRs) [51]. Meanwhile, metabiotics indirectly enhance digestive enzyme activity by regulating the composition of gut microbiota (such as increasing the abundance of lactic acid bacteria), inhibiting pathogen damage to the intestinal mucosa, and improving intestinal barrier function [52]. A healthy gut environment reduces energy consumption in inflammatory responses, allowing more metabolic resources to be used for digestive enzyme synthesis. Some metabiotic metabolites (such as bacteriocins and organic acids) can also protect digestive enzymes from degradation by regulating intestinal pH or inhibiting protease inhibitors [53]. The lipase content did not change significantly, which may be related to the differences in fat metabolism pathways between poultry and mammals. Fat digestion in chickens mainly depends on pancreatic lipase and bile salt-activated lipase (BSSL), while the regulatory effect of metabiotics on BSSL is limited. In the future, it may be considered to combine it with bile acid preparations to enhance the effect. In summary, metabiotics enhance the activity of trypsin and amylase in the jejunum through multiple pathways, which is of great significance for improving the digestion and absorption of carbohydrates and proteins in feed, and also provides new evidence for the use of *Lactobacillus reuteri* metabiotics to improve the feed utilization rate of broilers.

### 4.6. Effects of HSY and LE on the Jejunal Microbiota of Broilers

Disruption of gut microbiota balance can lead to excessive proliferation of harmful bacteria, which in turn affects gut health and nutrient absorption efficiency [54]. This study found through α-diversity analysis that HSY significantly increased the Chao1 index but had no significant effect on the Simpson and Shannon indices. This result indicates that metabiotics have a specific regulatory effect on gut microbiota, mainly by promoting the proliferation of specific beneficial bacteria to increase species richness. Inactivated bacteria and metabolites in metabiotics provide nutrient substrates for microorganisms (such as oligosaccharides and bacteriocins), while the short-chain fatty acids (such as butyric acid) produced by them create a favorable growth environment for symbiotic bacteria by regulating gut pH and inhibiting pathogen colonization [55]. This finding is consistent with previous research results on metabiotics increasing the Chao1 index without affecting evenness [56]. In terms of microbiota structure, the control group was dominated by *Lactobacillus* and *Lactobacillus*, which is consistent with the gut microbiota characteristics of healthy broilers. LE treatment led to a decrease in the relative abundance of *Lactobacillus*, while *Enterococcus* and *Faecalibacterium* increased, indicating that energy restriction disrupted microbiota homeostasis. It is noteworthy that the addition of HSY to the LE diet significantly increased the relative abundance of *Enterococcus* and *Clostridium butyricum*. Among them, *Clostridium butyricum*, as an important acid-producing bacterium, can produce a large amount of short-chain fatty acids, which play a positive role in maintaining intestinal health [57]. At the same time, the HSY treatment also enriched *Lactobacillus salivarius* and *Lactobacillus avium*, which have probiotic properties, further optimizing the microbial community structure. In contrast, previous studies have found that HSY increases the relative abundance of *Ligilactobacillus* and *Streptococcus* in the ileum [11], or increases the relative abundance of *Romboutsia* and *Bacteroidota* [28], thus remodeling the ileal microbiota. The regulatory effect of HSY on the jejunal and ileal microbiota may differ. In any case, this is the first time we have found that low-energy diets disrupt the balance of the gut microbiota in broilers, while HSY can improve the gut microecological environment by specifically enriching beneficial bacteria (such as *Clostridium butyricum* and *Lactobacillus salivarius*), which may be an important mechanism by which it alleviates the negative effects of low-energy diets.

## 5. Conclusions

HSY can improve gut health and mitigate the negative impact of low-energy treatment on broiler growth performance by increasing the content of endogenous enzymes in the jejunum, improving gut microbiota structure, and increasing the content of short-chain fatty acids in the jejunum. Most importantly, the beneficial effect of this HSY is equivalent to 70 kcal of metabolizable energy, which provides an important basis for the economical feed-saving breeding strategy for broilers.

## Figures and Tables

**Table 1:** Composition and nutrient levels of basal and low-energy diets.

Content (%)	Basal Diet	Low-Energy Diet
**Ingredients**		
Corn	47.625	49.035
Wheat flour	10.000	10.000
Duck oil (emulsified oil)	3.760	2.600
Soybean meal	25.520	25.270
Peanut meal	3.000	3.000
Corn gluten meal	3.000	3.000
MSG fermentation residue	1.000	1.000
Feather meal	1.500	1.500
Limestone	0.960	0.960
Dicalcium phosphate	0.510	0.510
Bone meal	1.000	1.000
Sodium chloride	0.200	0.200
Choline chloride	0.100	0.100
Lysine	0.570	0.570
DL-methionine	0.150	0.150
Liquid DL-methionine	0.200	0.200
Threonine	0.175	0.175
Valine	0.040	0.040
Phytase	0.015	0.015
Sodium bicarbonate	0.125	0.125
Sodium humate	0.150	0.150
Vitamin premix ^1^	0.200	0.200
Mineral premix ^2^	0.200	0.200
Total	100.000	100.000
**Analyzed nutrient levels** ^3^		
Metabolizable energy/(Mcal/kg)	3.27	3.20
Crude protein	23.29	23.31
Lysine	1.44	1.44
Methionine	0.49	0.49
Methoxycysteine	0.86	0.86
Threonine	1.04	1.04
Tryptophan	0.25	0.25
Calcium	0.92	0.92
Total phosphorus	0.67	0.67
Available phosphorus	0.41	0.41

^1^ Vitamin premix (provided per kilogram of feed) included the following substances: Vitamin A, 6000 IU; Vitamin D, 3000 IU; Vitamin K, 3.2 mg; Vitamin B1, 3 mg; Vitamin B2, 4.0 mg; Vitamin B12, 0.025 mg; Vitamin E, 44 IU; Biotin, 0.0325 mg; folic acid, 2.00 mg; pantothenic acid, 15 mg; Niacin, 15 mg. ^2^ Mineral premix (provided per kilogram of feed) included the following substances: Copper sulfate (copper content 25.50%), 10 mg; zinc sulfate (zinc content 22.75%), 100 mg; ferrous sulfate (iron content 20.10%), 80 mg; manganese sulfate (manganese content 22.80%), 100 mg; sodium selenite (selenium content 45.60%), 0.3 mg; potassium iodide (iodine content 76.45%), 0.7 mg. ^3^ Calculated values.

**Table 2:** Primer sequences used in the present study.

Gene Name	Forward (5′ → 3′)	Reverse (5′ → 3′)
*β-actin*	CAACACAGTGCTGTCTGGTGGTA	ATCGTACTCCTGCTTGCTGATCC
*IL-10*	CAGACCAGCACCAGTCATCA	TCCCGTTCTCATCCATCTTCTC
*IFN-γ*	AAAGCCGCACATCAAACACA	GCCATCAGGAAGGTTGTTTTTC
*MHC-II*	ATAAGGCGTGGGCTCAGTTC	GAATTCGGGCAGCCTCCATA
*TNF-α*	GAGCGTTGACTTGGCTGTC	AAGCAACAACCAGCTATGCAC
*TGF-β*	TCATCACCAGGACAGCGTTA	TGTGATGGAGCCATTCATGT
*Claudin-1*	CATACTCCTGGGTCTGGTTGGT	GACAGCCATCCGCATCTTCT
*ZO-1*	CTTCAGGTGTTTCTCTTCCTCCTC	CTGTGGTTTCATGGCTGGATC
*Occludin*	ACGGCAGCACCTACCTCAA	GGGCGAAGAAGCAGATGAG
*SGLT1*	GATGTGCGGATACCTGAAGC	AGGGATGCCAACATGACTGA
*AQP8*	CCTTTGGGCCAGCTGTGATA	CACTTCAGGAACAGGCGGAT
*TRPV6*	CTGTGCTCACGTCCTCTGTT	TGTTGCTGTGTGACAGATGGT
*KCNJ13*	ACACCACCTGCTCTGAACAC	TAGAGATCTCCTTAAGGCCACTTG
*SLC1A1*	TGGCAAGCTGTCTAACCTGG	GCTCGCAAACCAATCTTCCC
*ACC*	AATGGCAGCTTTGGAGGTGT	TCTGTTTGGGTGGGAGGTG
*LXRα*	CAAAGGGAATGAATGAGC	AGCCGAAGGGCAAACAC
*SREBF1*	GCCCTCTGTGCCTTTGTCTTC	ACTCAGCCATGATGCTTCTTC
*ACOT8*	CCACTCGCTTCACTGCTACTTCG	TGGCACATCTTCAGCTTGGATCTTG
*FABP4*	TTGCACAAAACCAATCAGCCT	AGGGCCTTCAGGGAGAATGT
*pepT1*	TACGCATACTGTCACCATCA	TCCTGAGAACGGACTGTAAT

*IL-10*: Interleukin 10; *IFN-γ*: interferon gamma; *MHC-II*: major histocompatibility complex class II; *TNF-α*: tumor necrosis factor alpha; *TGF-β*: transforming growth factor beta 1; *ZO-1*: tight junction protein 1; *SGLT1*: sodium/glucose cotransporter 1; *AQP8*: Aquaporin 8; *TRPV6*: transient receptor potential cation channel subfamily V member 6; *KCNJ13*: potassium inwardly rectifying channel subfamily J member 13; *SLC1A1*: solute carrier family 1 member 1; *ACC*: acetyl-CoA carboxylase alpha; *LXRα*: Liver X receptor alpha; *SREBF1*: sterol regulatory element-binding transcription factor 1; *ACOT8*: Acyl-CoA thioesterase 8; *FABP4*: fatty acid-binding protein 4; *pepT1*: solute carrier family 15 member 1.

**Table 3:** Effects of HSY and LE on growth performance of broilers aged 1–14 days.

Item	Group				*p* Value
	CTR	LE	HSY	LEHSY	
d 1 BW (g)	46.08 ± 0.08	46.17 ± 0.11	46.17 ± 0.11	46.00 ± 0.00	0.506
d 14 BW (g)	505.72 ± 15.06	507.17 ± 18.01	508.15 ± 17.73	503.55 ± 14.86	0.912
ADG (g)	32.81 ± 1.06	32.93 ± 1.27	32.97 ± 1.25	32.59 ± 1.06	0.855
ADFI (g)	38.52 ± 1.10	38.44 ± 1.75	38.57 ± 1.45	37.81 ± 1.29	0.528
FCR	1.17 ± 0.02	1.17 ± 0.02	1.17 ± 0.01	1.16 ± 0.01	0.163

CTR (basal diet); LE (CTR-70 kcal ME/kg); HSY (CTR + 0.5 kg/t HSY); LEHSY (LE + 0.5 kg/t HSY). All groups were fed with basal diet during d 1–d 14. BW, body weight; ADG, average daily gain; ADFI, average daily feed intake; FCR, feed conversion ratio.

**Table 4:** Effects of HSY and LE on growth performance of broilers aged 15–39 days.

Energy × Postbiotic		FCR	ADG (g)	ADFI (g)
CTR + 0		1.45 ^b^	86.38	126
LE + 0		1.50 ^a^	85.71	129.1
CTR + 0.5		1.45 ^b^	86.52	126.11
LE + 0.5		1.45 ^b^	85.47	124.76
SEM		0.005	0.473	0.718
Main effect				
Energy	CTR	1.46 ± 0.005 ^B^	86.45 ± 0.65	126.06 ± 0.89
	LE	1.48 ± 0.008 ^A^	85.59 ± 0.66	126.93 ± 1.18
Postbiotic	0	1.48 ± 0.008 ^A^	86.05 ± 0.61	127.55 ± 1.04
	0.5	1.46 ± 0.005 ^B^	86.00 ± 0.71	125.43 ± 1.01
*p* value				
Energy		0.010	0.366	0.545
Postbiotic		0.013	0.959	0.147
Energy*Postbiotic		0.013	0.842	0.129

^a,b^ Means within the same column without common superscripts differ significantly among the four treatment groups (*p* < 0.05). ^A,B^ Means within the same column without common superscripts indicate a significant main effect of HSY or LE (*p* < 0.05). Means sharing the same superscript letters are not significantly different (*p* > 0.05). SEM, standard error of the mean. (0) indicates no supplementation of *Lactobacillus reuteri* postbiotics, and (0.5) indicates 0.5 kg/t *Lactobacillus reuteri* postbiotics supplementation. CTR + 0 (Group CTR: basal diet); LE + 0 (Group LE: CTR-70 kcal ME/kg); CTR + 0.5 (Group HSY: CTR + 0.5 kg/t HSY); LE + 0.5 (Group LEHSY: LE + 0.5 kg/t HSY). ADG, average daily gain; ADFI, average daily feed intake; FCR, feed conversion ratio.

**Table 5:** Effects of HSY and LE on growth performance of broilers aged 1–39 days.

Energy × Postbiotic		d 39 BW (g)	FCR	ADG (g)	ADFI (g)
CTR + 0		2665.25	1.40	66.69	93.20
LE + 0		2649.88	1.42	65.84	93.45
CTR + 0.5		2671.23	1.39	66.38	92.16
LE + 0.5		2640.28	1.39	65.70	91.35
SEM		12.574	0.003	0.317	0.437
Main effect					
Energy	CTR	2668.24 ± 17.77	1.39 ± 0.004 ^B^	66.53 ± 0.47	92.68 ± 0.63
	LE	2645.08 ± 17.05	1.41 ± 0.005 ^A^	65.77 ± 0.41	92.40 ± 0.63
Postbiotic	0	2657.57 ± 17.16	1.41 ± 0.005 ^A^	66.26 ± 0.43	93.32 ± 0.54
	0.5	2655.75 ± 17.99	1.39 ± 0.003 ^B^	66.04 ± 0.47	91.76 ± 0.67
*p* value					
Energy		0.362	0.030	0.237	0.754
Postbiotic		0.943	0.001	0.727	0.080
Energy*Postbiotic		0.758	0.083	0.899	0.546

(0) indicates no supplementation of *Lactobacillus reuteri* postbiotics, and (0.5) indicates 0.5 kg/t *Lactobacillus reuteri* postbiotics supplementation. ^A,B^ Means within the same column without common superscripts indicate a significant main effect of HSY or LE (*p* < 0.05). Means sharing the same superscript letters are not significantly different (*p* > 0.05). SEM, standard error of the mean. CTR + 0 (Group CTR: basal diet); LE + 0 (Group LE: CTR-70 kcal ME/kg); CTR + 0.5 (Group HSY: CTR + 0.5 kg/t HSY); LE + 0.5 (Group LEHSY: LE + 0.5 kg/t HSY). BW, body weight; ADG, average daily gain; ADFI, average daily feed intake; FCR, feed conversion ratio.

**Table 6:** Effects of HSY and LE on European performance efficiency factor of broilers.

Energy × Postbiotic		d 21	d 28	d 39
CTR + 0		381.77	430.47	476.67
LE + 0		362.58	418.00	454.18
CTR + 0.5		360.32	420.89	467.29
LE + 0.5		356.84	414.06	462.42
SEM		2.372	2.315	3.002
Main effect				
Energy	CTR	371.04 ± 3.94 ^A^	425.68 ± 3.51 ^A^	471.98 ± 4.72 ^A^
	LE	359.71 ± 3.37 ^B^	416.03 ± 3.06 ^B^	458.30 ± 3.72 ^B^
Postbiotic	0	372.18 ± 3.60 ^A^	424.24 ± 2.99	465.42 ± 4.75
	0.5	358.58 ± 3.57 ^B^	417.48 ± 3.71	464.86 ± 4.21
*p* value				
Energy		0.021	0.043	0.028
Postbiotic		0.006	0.151	0.925
Energy*Postbiotic		0.105	0.545	0.149

^A,B^ Means within the same column without common superscripts indicate a significant main effect of HSY or LE (*p* < 0.05). Means sharing the same superscript letters are not significantly different (*p* > 0.05). SEM, standard error of the mean. (0) indicates no supplementation of *Lactobacillus reuteri* postbiotics, and (0.5) indicates 0.5 kg/t *Lactobacillus reuteri* postbiotics supplementation. CTR + 0 (Group CTR: basal diet); LE + 0 (Group LE: CTR-70 kcal ME/kg); CTR + 0.5 (Group HSY: CTR + 0.5 kg/t HSY); LE + 0.5 (Group LEHSY: LE + 0.5 kg/t HSY).

**Table 7:** Effects of HSY and LE on serum biochemical parameters of broilers.

Energy × Postbiotic		TP	ALB	AST	ALT	TC	TG	GLU	CA	P	CREA	HDL	LDL	UA	CK	TB	DB	LDH
CTR + 0		38.47	11.92	301.94	4.44	3.20	0.72	13.67	3.72	2.51	9.36	2.09	0.55	489.21	13,897.11	14.12	1.13	1588.71
CTR + 0.5		37.47	11.23	379.82	4.73	3.27	0.78	13.82	3.54	2.36	9.09	2.18	0.57	437.82	16,926.45	16.60	1.24	1826.26
LE + 0		36.37	11.70	284.40	4.82	3.17	0.70	13.79	3.46	2.14	10.10	2.09	0.51	427.82	9837.59	15.35	0.78	1250.50
LE + 0.5		40.25	11.43	390.22	5.33	3.28	0.66	12.51	3.59	2.12	10.10	2.23	0.53	457.09	16,395.89	16.37	0.73	1535.64
SEM		0.799	0.178	13.951	0.176	0.064	0.032	0.305	0.043	0.049	0.23	0.045	0.022	17.987	681.475	0.451	0.048	73.564
Main effect																		
Energy	CTR	37.42± 0.100	11.81± 0.266	293.17± 13.06 ^B^	4.63± 0.215	3.19± 0.081	0.71± 0.042	13.73± 0.252	3.59± 0.063	2.33± 0.083	9.73± 0.351	2.09± 0.050	0.53± 0.029	458.51 ± 21.60	11,867.35± 890.52 ^B^	14.74± 0.634	0.95± 0.070	1419.60± 103.06
	LE	38.86± 1.033	11.33± 0.191	385.02± 22.72 ^A^	5.03± 0.248	3.28± 0.078	0.72± 0.040	13.16± 0.474	3.57± 0.057	2.24± 0.048	9.59± 0.328	2.21± 0.064	0.55± 0.025	447.46 ± 23.90	16,661.16± 950.98 ^A^	16.49± 0.679	0.99± 0.078	1680.95± 100.94
Postbiotic	0	37.97 ± 0.844	11.58 ± 0.233	340.88 ± 17.93	4.59± 0.234	3.24± 0.077	0.75± 0.044	13.75 ± 0.479	3.63± 0.069	2.43± 0.077 ^A^	9.22± 0.319	2.14 ± 0.059	0.56 ± 0.027	463.51 ± 23.79	15,411.78 ± 997.81	15.36 ± 0.769	1.18 ± 0.071 ^B^	1707.48 ± 100.52 ^A^
	0.5	38.31 ± 1.172	11.56 ± 0.236	337.31 ± 23.39	5.08 ± 0.222	3.23 ± 0.082	0.68 ± 0.037	13.15 ± 0.273	3.53 ± 0.045	2.13 ± 0.039 ^B^	10.10 ± 0.307	2.16 ± 0.059	0.52 ± 0.026	442.46 ± 22.15	13,116.74 ± 956.40	15.86 ± 0.574	0.75 ± 0.044 ^A^	1393.07 ± 101.83 ^B^
*p* value																		
Energy		0.373	0.191	0.002	0.264	0.485	0.883	0.358	0.759	0.373	0.767	0.208	0.539	0.760	0.001	0.060	0.718	0.084
Postbiotic		0.833	0.971	0.899	0.172	0.938	0.288	0.335	0.222	0.004	0.066	0.843	0.334	0.562	0.101	0.582	<0.001	0.039
Energy*Postbiotic		0.136	0.567	0.620	0.743	0.912	0.469	0.251	0.0861	0.497	0.780	0.802	0.964	0.270	0.204	0.423	0.405	0.872

^A,B^ Means within the same column without common superscripts indicate a significant main effect of HSY or LE (*p* < 0.05). Means sharing the same superscript letters are not significantly different (*p* > 0.05). SEM, standard error of the mean. (0) indicates no supplementation of *Lactobacillus reuteri* postbiotics, and (0.5) indicates 0.5 kg/t *Lactobacillus reuteri* postbiotics supplementation. CTR + 0 (Group CTR: basal diet); LE + 0 (Group LE: CTR-70 kcal ME/kg); CTR + 0.5 (Group HSY: CTR + 0.5 kg/t HSY); LE + 0.5 (Group LEHSY: LE + 0.5 kg/t HSY). TP, total protein (g/dL); ALB, albumin (g/dL); AST, aspartate aminotransferase (U/L); ALT, alanine aminotransferase (U/L); TC, total cholesterol (mmol/L); TG, triglyceride (mmol/L); GLU, glucose (mmol/L); CA, calcium (nmol/L); P, phosphorus (nmol/L); CREA, creatinine (mg/dL); HDL, high-density lipoprotein (mmol/L); LDL, low-density lipoprotein (mmol/L); UA, uric acid (mmol/L); CK, creatine kinase (U/L); TB, total bilirubin (μmol/L); DB, direct bilirubin (μmol/L); LDH, lactate dehydrogenase (U/L).

**Table 8:** Effects of HSY and LE on the intestinal index of broilers.

Energy × Postbiotic		Duodenum (cm/kg)	Jejunum (cm/kg)	Ileum (cm/kg)	Cecum (cm/kg)
CTR + 0		5.96	27.00	28.10	6.94
LE + 0		5.96	28.01	27.52	6.80
CTR + 0.5		5.75	28.00	27.36	7.24
LE + 0.5		5.91	27.88	27.15	7.07
SEM		0.083	0.433	0.468	0.099
Main effect					
Energy	CTR	5.86 ± 0.12	27.50 ± 0.69	27.73 ± 0.67	7.09 ± 0.15
	LE	5.94 ± 0.11	27.94 ± 0.50	27.34 ± 0.64	6.94 ± 0.13
Postbiotic	0	5.96 ± 0.11	27.50 ± 0.61	27.81 ± 0.69	6.87 ± 0.16
	0.5	5.83 ± 0.12	27.94 ± 0.60	27.25 ± 0.61	7.16 ± 0.11
*p* value					
Energy		0.625	0.610	0.679	0.439
Postbiotic		0.434	0.619	0.556	0.150
Energy*Postbiotic		0.628	0.519	0.847	0.932

SEM, standard error of the mean. (0) indicates no supplementation of *Lactobacillus reuteri* postbiotics, and (0.5) indicates 0.5 kg/t *Lactobacillus reuteri* postbiotics supplementation. CTR + 0 (Group CTR: basal diet); LE + 0 (Group LE: CTR-70 kcal ME/kg); CTR + 0.5 (Group HSY: CTR + 0.5 kg/t HSY); LE + 0.5 (Group LEHSY: LE + 0.5 kg/t HSY).

**Table 9:** Effects of HSY and LE on intestinal morphology of broilers.

Energy × Postbiotic		VH (μm)	CD (μm)	VH/CD	VA (μm^2^)
CTR + 0		859.55	178.81	4.78	107,187.69
LE + 0		971.59	160.33	6.27	134,878.41
CTR + 0.5		996.05	189.69	5.27	145,090.66
LE + 0.5		1015.74	177.11	5.71	135,659.74
SEM		42.474	4.14	0.206	4809.834
Main effect					
Energy	CTR	996.05 ± 56.97	189.69 ± 4.40	5.27 ± 0.28	145,090.66 ± 7378.54 ^A^
	LE	993.67 ± 60.66	168.72 ± 7.01	5.99 ± 0.30	135,269.07 ± 7562.97 ^B^
Postbiotic	0	915.57 ± 53.09	169.57 ± 5.66	5.52 ± 0.31 ^A^	121,033.05 ± 7628.78
	0.5	1005.90 ± 65.61	183.40 ± 6.21	5.49 ± 0.30 ^B^	140,375.20 ± 6387.76
*p* value					
Energy		0.295	0.104	0.930	0.052
Postbiotic		0.443	0.069	0.025	0.349
Energy*Postbiotic		0.590	0.724	0.212	0.062

^A,B^ Means within the same column without common superscripts indicate a significant main effect of HSY or LE (*p* < 0.05). Means sharing the same superscript letters are not significantly different (*p* > 0.05). SEM, standard error of the mean. (0) indicates no supplementation of *Lactobacillus reuteri* postbiotics, and (0.5) indicates 0.5 kg/t *Lactobacillus reuteri* postbiotics supplementation. CTR + 0 (Group CTR: basal diet); LE + 0 (Group LE: CTR-70 kcal ME/kg); CTR + 0.5 (Group HSY: CTR + 0.5 kg/t HSY); LE + 0.5 (Group LEHSY: LE + 0.5 kg/t HSY). VH, villus height; CD, crypt depth; VA, villus area.

**Table 10:** Effects of HSY and LE on the mRNA expression of the intestinal mucosal barrier, nutrient transport, immunity, and lipid metabolism genes of broilers.

Energy × Postbiotic		*Intestinal Mucosal Barrier*			*Nutrient Transport*						*Immunity*					*Lipid Metabolism*				
		*Claudin-1*	*ZO-1*	*Occludin*	*SGLT1*	*AQP-8*	*TRPV6*	*KCNJ13*	*SLC1A1*	*pepT1*	*IFN-γ*	*IL-10*	*MHC-II*	*TNF-α*	*TGF-β*	*ACC*	*LXRα*	*SREBF1*	*ACOT8*	*FABP4*
CTR + 0		1.00	1.00	1.00 ^b^	1.00	1.00	1.00 ^b^	1.00 ^b^	1.00 ^bc^	1.00 ^a^	1.00	1.00 ^b^	1.00	1.00	1.00	1.00	1.00	1.00	1.00 ^c^	1.00
CTR + 0.5		1.63	0.94	2.11 ^a^	1.81	1.27	0.76 ^b^	2.67 ^a^	1.94 ^a^	0.90 ^a^	1.19	0.63 ^c^	1.4	0.91	0.95	0.75	1.02	0.70	1.51 ^b^	1.87
LE + 0		1.61	1.03	1.14 ^b^	0.87	1.50	0.73 ^b^	2.32 ^a^	1.32 ^b^	0.18 ^b^	1.45	1.03 ^b^	1.08	0.97	1.08	0.82	0.65	0.80	1.96 ^a^	2.27
LE + 0.5		1.73	1.02	0.84 ^b^	0.76	1.13	3.37 ^a^	1.44 ^b^	0.83 ^c^	0.71 ^a^	1.41	1.45 ^a^	1.35	0.95	1.33	0.60	0.61	0.67	1.03 ^c^	2.49
SEM		0.063	0.039	0.054	0.072	0.102	0.097	0.112	0.079	0.076	0.055	0.04	0.049	0.026	0.044	0.033	0.052	0.035	0.066	0.099
Main effect																				
Energy	CTR	1.31 ± 0.091	1.01 ± 0.055	1.07 ± 0.064	0.93 ± 0.066	1.25 ± 0.184	0.87 ± 0.137 ^B^	1.66 ± 0.191	1.16 ± 0.086	0.59 ± 0.105	1.23 ± 0.092	1.04 ± 0.048	1.04 ± 0.065 ^B^	0.98 ± 0.032	1.04 ± 0.057	0.91 ± 0.054 ^A^	0.83 ± 0.075	0.90 ± 0.060 ^A^	1.48 ± 0.147	1.64 ± 0.181 ^B^
	LE	1.68 ± 0.105	0.98 ± 0.055	1.47 ± 0.158	1.28 ± 0.166	1.20 ± 0.093	2.07 ± 0.304 ^A^	2.05 ± 0.216	1.38 ± 0.176	0.8 ± 0.137	1.3 ± 0.081	1.03 ± 0.113	1.37 ± 0.072 ^A^	0.93 ± 0.039	1.14 ± 0.079	0.67 ± 0.041 ^B^	0.81 ± 0.088	0.69 ± 0.039 ^B^	1.27 ± 0.087	2.18 ± 0.163 ^A^
Postbiotic	0	1.32 ± 0.103	0.97 ± 0.055	1.55 ± 0.146 ^A^	1.40 ± 0.150 ^A^	1.14 ± 0.094	0.88 ± 0.130 ^B^	1.84 ± 0.247	1.47 ± 0.165	0.95 ± 0.120 ^A^	1.10 ± 0.082 ^B^	0.83 ± 0.059 ^B^	1.2 ± 0.082	0.95 ± 0.037	0.97 ± 0.052 ^B^	0.87 ± 0.059 ^A^	1.01 ± 0.095 ^A^	0.85 ± 0.060	1.25 ± 0.101	1.44 ± 0.169 ^B^
	0.5	1.67 ± 0.094	1.02 ± 0.054	0.99 ± 0.065 ^B^	0.81 ± 0.068 ^B^	1.32 ± 0.182	2.05 ± 0.309 ^A^	1.88 ± 0.159	1.07 ± 0.094	0.45 ± 0.104 ^B^	1.43 ± 0.071 ^A^	1.45 ± 0.082 ^A^	1.21 ± 0.075	0.96 ± 0.004	1.20 ± 0.074 ^A^	0.71 ± 0.042 ^B^	0.63 ± 0.035 ^B^	0.74 ± 0.047	1.49 ± 0.136	2.38 ± 0.133 ^A^
*p* value																				
Energy		0.005	0.698	0.001	0.019	0.800	<0.001	0.084	0.163	0.17	0.5	0.79	0.002	0.287	0.259	0.001	0.880	0.004	0.108	0.009
Postbiotic		0.007	0.511	<0.001	<0.001	0.378	<0.001	0.858	0.016	0.002	0.004	<0.001	0.874	0.907	0.013	0.015	0.001	0.120	0.073	<0.001
Energy*Postbiotic		0.047	0.756	<0.001	0.003	0.121	<0.001	<0.001	<0.001	0.046	0.296	<0.001	0.509	0.472	0.088	0.811	0.757	0.235	<0.001	0.105

^a,b,c^ Means within the same column without common superscripts differ significantly among the four treatment groups (*p* < 0.05). ^A,B^ Means within the same column without common superscripts indicate a significant main effect of HSY or LE (*p* < 0.05). Means sharing the same superscript letters are not significantly different (*p* > 0.05). SEM, standard error of the mean. (0) indicates no supplementation of *Lactobacillus reuteri* postbiotics, and (0.5) indicates 0.5 kg/t *Lactobacillus reuteri* postbiotics supplementation. CTR + 0 (Group CTR: basal diet); LE + 0 (Group LE: CTR-70 kcal ME/kg); CTR + 0.5 (Group HSY: CTR + 0.5 kg/t HSY); LE + 0.5 (Group LEHSY: LE + 0.5 kg/t HSY). *IL-10*: Interleukin 10; *IFN-γ*: interferon gamma; *MHC-II*: major histocompatibility complex class II; *TNF-α*: tumor necrosis factor alpha; *TGF-β*: transforming growth factor beta 1; *ZO-1*: tight junction protein 1; *SGLT1*: sodium/glucose cotransporter 1; *AQP8*: Aquaporin 8; *TRPV6*: transient receptor potential cation channel subfamily V member 6; *KCNJ13*: potassium inwardly rectifying channel subfamily J member 13; *SLC1A1*: solute carrier family 1 member 1; *ACC*: acetyl-CoA carboxylase alpha; *LXRα*: Liver X receptor alpha; *SREBF1*: sterol regulatory element-binding transcription factor 1; *ACOT8*: Acyl-CoA thioesterase 8; *FABP4*: fatty acid-binding protein 4; *pepT1*: solute carrier family 15 member 1.

**Table 11:** Effect of HSY and LE on short-chain fatty acids in the jejunum of broilers.

Energy × Postbiotic		Acetic Acid (ug/g)	Propionic Acid (ug/g)	Butyric Acid (ug/g)
CTR + 0		856.22	252.14 ^c^	180.48
LE + 0		952.90	509.37 ^a^	346.47
CTR + 0.5		990.08	422.63 ^b^	271.05
LE + 0.5		1206.23	481.36 ^a^	364.71
SEM		48.895	16.073	9.153
Main effect				
Energy	CTR	923.15 ± 51.66	337.39 ± 26.22 ^B^	225.76 ± 16.12 ^B^
	LE	1079.56 ± 59.04	495.37 ± 37.13 ^A^	355.59 ± 21.57 ^A^
Postbiotic	0	904.56 ± 60.35	380.76 ± 50.27 ^B^	263.47 ± 27.23 ^B^
	0.5	1098.15 ± 62.24	451.99 ± 17.38 ^A^	317.8 b ± 20.00 ^A^
*p* value				
Energy		0.120	0.001	0.001
Postbiotic		0.057	0.034	0.006
Energy*Postbiotic		0.546	0.004	0.057

^a,b,c^ Means within the same column without common superscripts differ significantly among the four treatment groups (*p* < 0.05). ^A,B^ Means within the same column without common superscripts indicate a significant main effect of HSY or LE (*p* < 0.05). Means sharing the same superscript letters are not significantly different (*p* > 0.05). SEM, standard error of the mean. (0) indicates no supplementation of *Lactobacillus reuteri* postbiotics, and (0.5) indicates 0.5 kg/t *Lactobacillus reuteri* postbiotics supplementation. CTR + 0 (Group CTR: basal diet); LE + 0 (Group LE: CTR-70 kcal ME/kg); CTR + 0.5 (Group HSY: CTR + 0.5 kg/t HSY); LE + 0.5 (Group LEHSY: LE + 0.5 kg/t HSY).

**Table 12:** Effect of HSY and LE on jejunal lysozyme of broilers.

Energy × Postbiotic		Amylase (U/g Prot)	Lipase (U/mg Prot)	Trypsin (U/mg Prot)
CTR + 0		0.01 ^b^	10.58	258.12
LE + 0		0.02 ^b^	13.62	312.67
CTR + 0.5		0.04 ^a^	10.40	1001.85
LE + 0.5		0.02 ^ab^	8.20	901.36
SEM		0.002	0.713	50.705
Main effect				
Energy	CTR	0.02 ± 0.004	10.49 ± 0.98	629.98 ± 117.39
	LE	0.02 ± 0.002	10.91 ± 1.18	607.02 ± 87.63
Postbiotic	0	0.02 ± 0.004 ^B^	12.10 ± 1.10	285.40 ± 20.18 ^B^
	0.5	0.03 ± 0.004 ^A^	9.30 ± 0.93	951.60 ± 102.26 ^A^
*p* value				
Energy		0.670	0.772	0.822
Postbiotic		0.005	0.062	<0.001
Energy*Postbiotic		0.047	0.079	0.450

^a,b^ Means within the same column without common superscripts differ significantly among the four treatment groups (*p* < 0.05). ^A,B^ Means within the same column without common superscripts indicate a significant main effect of HSY or LE (*p* < 0.05). Means sharing the same superscript letters are not significantly different (*p* > 0.05). SEM, standard error of the mean. (0) indicates no supplementation of *Lactobacillus reuteri* postbiotics, and (0.5) indicates 0.5 kg/t *Lactobacillus reuteri* postbiotics supplementation. CTR + 0 (Group CTR: basal diet); LE + 0 (Group LE: CTR-70 kcal ME/kg); CTR + 0.5 (Group HSY: CTR + 0.5 kg/t HSY); LE + 0.5 (Group LEHSY: LE + 0.5 kg/t HSY).

**Table 13:** Effect of HSY and LE on alpha diversity of jejunal microbiota of broilers.

Energy × Postbiotic		Simpson	Chao1	Shannon
CTR + 0		0.57	39.51	1.77
LE + 0		0.61	51.76	1.79
CTR + 0.5		0.51	55.99	1.76
LE + 0.5		0.61	55.23	1.91
SEM		0.026	1.813	0.077
Main effect				
Energy	CTR	0.54 ± 0.040	47.75 ± 4.92	1.76 ± 0.14
	LE	0.61 ± 0.042	53.50 ± 16.40	1.85 ± 0.170
Postbiotic	0	0.59 ± 0.047	45.64 ± 16.91 ^B^	1.78 ± 0.18
	0.5	0.56 ± 0.032	55.61 ± 2.78 ^A^	1.83 ± 0.13
*p* value				
Energy		0.171	0.120	0.588
Postbiotic		0.537	0.009	0.735
Energy*Postbiotic		0.566	0.080	0.676

^A,B^ Means within the same column without common superscripts indicate a significant main effect of HSY or LE (*p* < 0.05). Means sharing the same superscript letters are not significantly different (*p* > 0.05). SEM, standard error of the mean. (0) indicates no supplementation of *Lactobacillus reuteri* postbiotics, and (0.5) indicates 0.5 kg/t *Lactobacillus reuteri* postbiotics supplementation. CTR + 0 (Group CTR: basal diet); LE + 0 (Group LE: CTR-70 kcal ME/kg); CTR + 0.5 (Group HSY: CTR + 0.5 kg/t HSY); LE + 0.5 (Group LEHSY: LE + 0.5 kg/t HSY).

## Data Availability

Jejunal chyme 16S rRNA data has been transmitted to the sequence read archive (PRJNA1414861).
